# Broadening Bandwidths of Few-Layer Absorbers by Superimposing Two High-Loss Resonators

**DOI:** 10.1186/s11671-020-03471-1

**Published:** 2021-02-10

**Authors:** Dong Wu, Jianjun Chen

**Affiliations:** 1 State Key Laboratory for Mesoscopic Physics, School of Physics, Peking University, Beijing, 100871 China; 2 Department of Physics and Applied Optics Beijing Area Major Laboratory, Beijing Normal University, Beijing, 100875 China; 3 Peking University Yangtze Delta Institute of Optoelectronics, Nantong, 226010 Jiangsu China; 4 Frontiers Science Center for Nano-Optoelectronics & Collaborative Innovation Center of Quantum Matter, Peking University, Beijing, 100871 China; 5 Collaborative Innovation Center of Extreme Optics, Shanxi University, Taiyuan, 030006 Shanxi China

**Keywords:** Absorption, Metasurface, Solar energy, Non-noble metal, Resonance

## Abstract

Efficient broadband absorption of solar radiation is desired for sea water desalination, icephobicity and other renewable energy applications. We propose an idea of superimposing two high-loss resonances to broaden bandwidths of a few-layer absorber, which is made of dielectric/ metal/dielectric/ metal layers. Both the simulation and experiment show that the structure has an averaged absorption efficiency higher than 97% at wavelengths ranging from 350 to 1200 nm. The bandwidth of the absorption larger than 90% is up to 1000 nm (410–1410 nm), which is greater than that (≤ 750 nm) of previous MIM planar absorbers. Especially, the average absorption from 350 to 1000 nm is kept above 90% at an incidence angle as high as 65°, meanwhile still maintained above 80% even at an incident angle of 75°. The performance of angular insensitivity is much better than that of previous few-layer solar absorbers. The flexible 1D nonoble metasurface absorbers are fabricated in a single evaporation step. Under the illumination of a halogen lamp of *P* = 1.2 kW/m^2^, the flexible metasurface increases its surface temperature by 25.1 K from room temperature. Further experiments demonstrate that the heat localization rapidly melts the accumulated ice. Our illumination intensity (*P* = 1.2 kW/m^2^) is only half of that (*P* = 2.4 kW/m^2^) in previous solar anti-ice studies based on gold/TiO_2_ particle metasurfaces, indicating that our metasurface is more advantageous topractical applications. Our results illustrate an effective pathway toward the broadband metasurface absorbers with the attractive properties of mechanical flexibility, low cost of the no-noble metals, and large-area fabrications, which have promising prospects in the applications of solar heat utilization.

## Introduction

An optical absorber with a high and broad absorption has long been a major scientific and technological goals [1–9] for many applications, including thermal photovoltaic [10–15], steam generation [16, 17], and photodetection [18]. In recent years, the optical metamaterial/metasurface absorbers, artificially structured materials made from 2D arrays of subwavelength unit-cells, have been widely investigated and developed [1, 2], such as densely packed nanowires [19], nanotubes [15], tapered grooves [20–22], and pyramidal designs [23, 24]. Although enormous efforts are made in performance enhancements of these absorbers based on 2D arrays [25–37], the fabrication complexity of most these nanostructures, requiring electron beam lithography (EBL) [20], focused ion beam (FIB) milling [23], Nanoimprint lithography [22], or Lithography technology [24], hinders their further upscaling.

For solving these problems, 1D metasurfaces based on the concept of lithography-free planar designs become a topic of intensive investigations in recent years [1, 5, 8, 25–27]. Recently, scientists proved the absorption capability of some few-layer configurations (such as single noble metal layer, insulator–metal (IM), and metal–insulator-metal (MIM) structure) [1, 8, 25–27, 38–48], which are favorable to local accumulation of absorbed heat. Firstly, for the simple planar configurations based on noble metals (such as Au and Ag), the absorption bandwidths (*A* > 90%) are smaller than 500 nm because the absorption is caused by only a surface plasmon polaritons (SPP) effect mechanism [1–8]. These absorbers based on the SPP effect also show innately angle-dependent property due to the momentum matching conditions [1–8]. Besides, some absorbers using noble metals based on IM or MIM planar configuration were also proposed and demonstrated by using Fabry–Perot (FP) resonance. However, for these planar absorbers (such as Ge/Au [48] and Ag/Si/Ag [49]), the absorption bandwidths (*A* > 80%) are generally less than 300 nm owing to the utilization of only one FP resonance. Meanwhile, the material cost of noble metal in most of the above-mentioned absorbers is expensive [1–8, 48, 50]. Recently, several groups used non-noble metals (such as Mo or Gr) based on MIM planar nanostructures to demonstrate optical absorbers [50, 51]. The Mo/Al_2_O_3_/Mo absorber based on a single Febry–Perot (FP) resonance showed absorption above 90% from 400 to 900 nm [50]. The Cr/Al_2_O_3_/Cr absorber based on one FP resonance showed absorption above 90% from 400 to 1150 nm [51]. For most of the reported few-layer planar absorbers, the bandwidth ∆*λ*_BW_ (A > 90%) in the visible-near infrared wavelengths is smaller than 750 nm. Meanwhile, for these MIM planar nanostructures based on one FP resonance, the average absorption efficiency at wavelengths of 400–1000 nm would drop below 90% for an incident angle greater than 40° under a TE–polarization incidence. Such angle dependent spectral characteristic is a significant drawback, which makes the absorbers difficult to be applicable in practical usages. Thus, designing and realizing few-layer non-noble 1D metasurfaces to achieve omnidirectional, broadband, and efficient absorption are challenging but necessary for practical applications.

Here, we propose and experimentally demonstrate a few-layer non-noble 1D metasurface, which superimposes two high-loss resonators to broaden bandwidths (∆*λ*_BW_) of absorbers. The few-layer non-noble 1D metasurface is dielectric/metal/dielectric thin layers on a thick metal film, and it comprises of two high-loss resonators. Due to the superimposing of the two high-loss resonators, the average absorption efficiency of our proposed metasurface is above 97% at wavelengths from 400 to 1200 nm. The absorption bandwidths (*A* > 90%) is up to 1000 nm (410–1410 nm), which is greater than that (∆*λ*_BW_ = 750 nm[51]) of previous MIM planar absorbers [1–8, 48, 50]. Moreover, the average absorption for a wide range of incident angles up to 0–65° all surpasses 90% at wavelengths ranging from 350 to 1000 nm. This makes our absorbers more beneficial for practical applications compared to previous MIM planar absorbers [1–8, 48, 50], of which the average absorption efficiency at wavelengths of 400–1000 nm would drop below 90% for an incident angle larger than 40° under TE-polarization incidence. The metasurface is fabricated by a single step of electron beam vapor deposition on a glass substrate as well as a flexible PET substrate. The measured absorption spectra of the non-noble metasurface match well with the simulation results. Due to the efficient optical absorption and photothermal energy conversion in the ultra-thin absorption layer (thickness = 10 nm), the non-noble metasurface exhibits a temperature increase (ΔTe = 25.1 K) when it is illuminated by a halogen light source (*P* = 1.2 kW/m^2^). The increased temperature (ΔTe = 25.1 K) is higher than that of the recently reported solar absorbers based on a gold-particle metasurface (ΔTe = 12 °C under *P* = 2.4 kW/m^2^) [48] and gold/nickel plasmonic metasurface (ΔTe = 8 °C under *P* = 1.2 kW/m^2^) [49]. For practical applications, we demonstrate that the metasurface is capable of removing ice under a halogen light source (*P* = 1.2 kW/m^2^). This is more efficient compared to the previous solar anti-ice work based on a gold/TiO_2_ particle metasurface using a halogen light source with *P* = 2.4 kW/m^2^ [48]. The lithography-free fabrication of our 1D few-layer metasurface is easy to scale, facilitating its extensive use in practical photo-thermal applications.

## Design and Methods

**Fig. 1:**
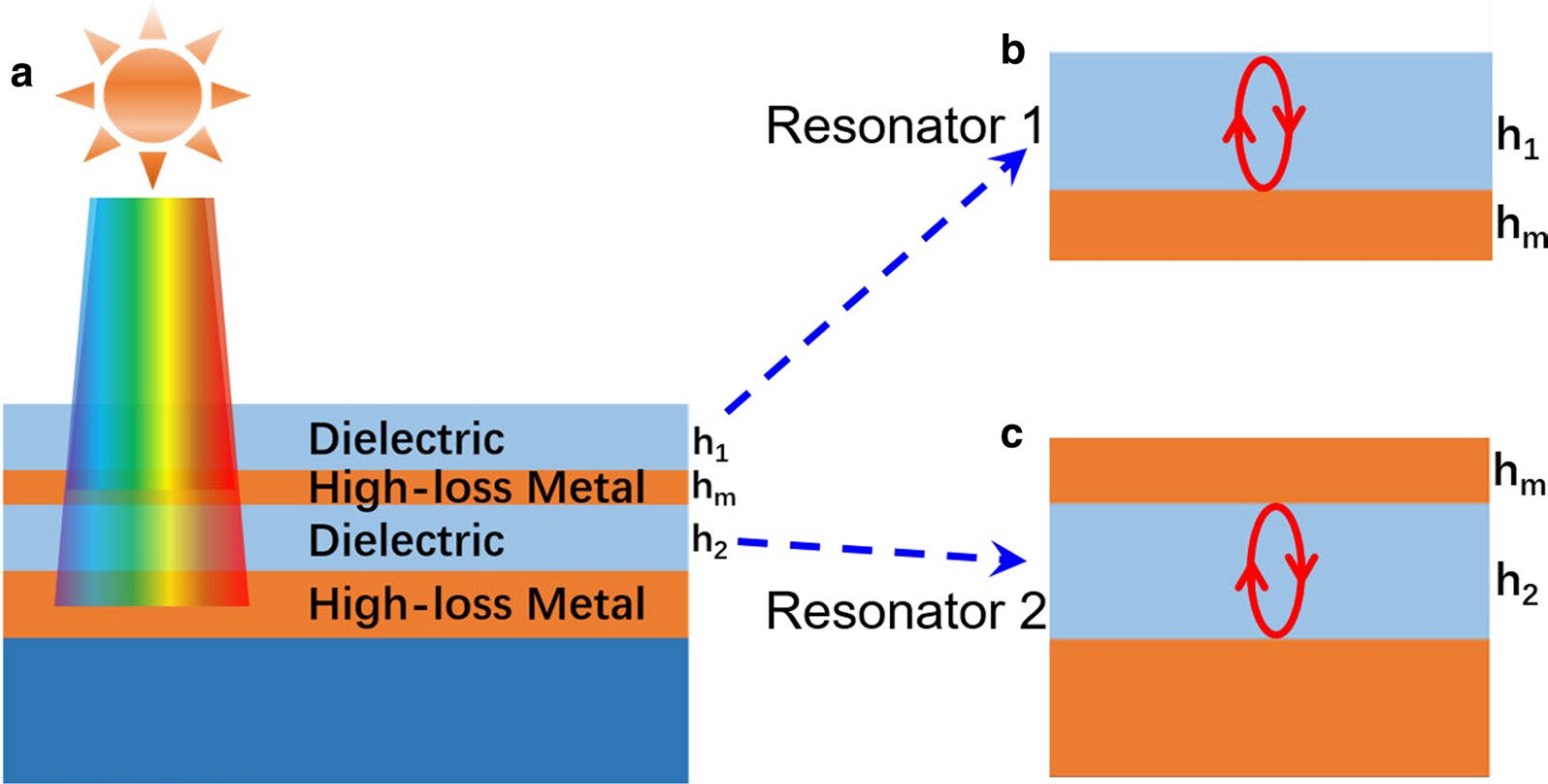
Design of 1D few-layer metasurfaces

The designed 1D few-layer metasurface is consisting of insulator/metal (high-loss)/insulator thin layers on a thick metal film, as shown in Fig. 1a. The thicknesses of the top three thin layers are *h*_1_, *h*_m_, and, *h*_2_, respectively. The illuminating light can be reflected back and forth off the dielectric-air interface and the dielectric-metal interface in the IM planar nanostructure, constructing a resonator [48], as shown in Fig. 1b (Resonator 1). The length of Resonator 1 is *h*_*1*_. Similarly, the metal (high-loss)/insulator/metal (high-loss) planar nanostructure is also a resonator [49–51] (denoted by Resonator 2 in Fig. 1c), and the length of Resonator 2 is h_2_. The resonant condition of the two resonators is

1 $$2\left( {\frac{2\pi }{{{\lambda_{{\text{res}}}}}}} \right){n_i}{t_i} + {\emptyset_b} + {\emptyset_t} = 2\pi m$$

Herein, *λ*_res_ is the resonance wavelength. *n*_i_ and *t*_i_ are the refractive index and thickness of the insulator layer, respectively. *m* is an integer number that determines the order of the resonant mode. Φ_*b*_ and Φ_*t*_ are the phase shift acquired from two reflections. Based on Eq. (1), by increasing *t*_*i*_, the resonant wavelength *λ*_*res*_ will red-shift. Besides, with the increase of the thickness (*t*_*i*_) of the insulator layer, the number of resonant modes will increase. To increase the absorption and broaden the operation bandwidth (∆*λ*_BW_) of resonators, high-loss metal materials are employed for both the top and bottom metal layers. As we all know, there are many high-loss materials in nature, such as Ti, W, and Ni. These materials are inexpensive. Herein, Ti is chosen as the high-loss metal (the second layer and fourth layer). An MgF_2_ layer is chosen as the first and third layer. Other similar dielectrics such as SiO_2_, TiO_2_, and polymers can also be used as the dielectric layers.

To prove that the structure in Fig. 1a has two resonators, the absorption spectra of the IM and MIM planar structures in Fig. 1b, c are simulated and depicted, respectively. The absorption of the metasurface can be calculated using a formula of *A* = 1 − *R* − *T*. The two-dimensional finite-difference time-domain (FDTD) method is performed to simulate the proposed structure. A normally incident light is incident along the negative z-direction with the polarization along the x-direction. The mesh size is set to be 1 nm. Periodic boundary conditions are applied in the x and y directions. Perfectly matched layers (PML) are implemented at the upper and bottom boundary of the model. For the permittivity values of dielectric and metal materials, the experimental data in[53] are employed. In the experiment, the designed metasurface is fabricated by using an E-beam evaporator. The optical transmission (T) and reflection (R) spectra of the metasurface are measured by a Shimadzu UV3600 spectrophotometer.

## Simulation Results and Discussion

**Fig. 2:**
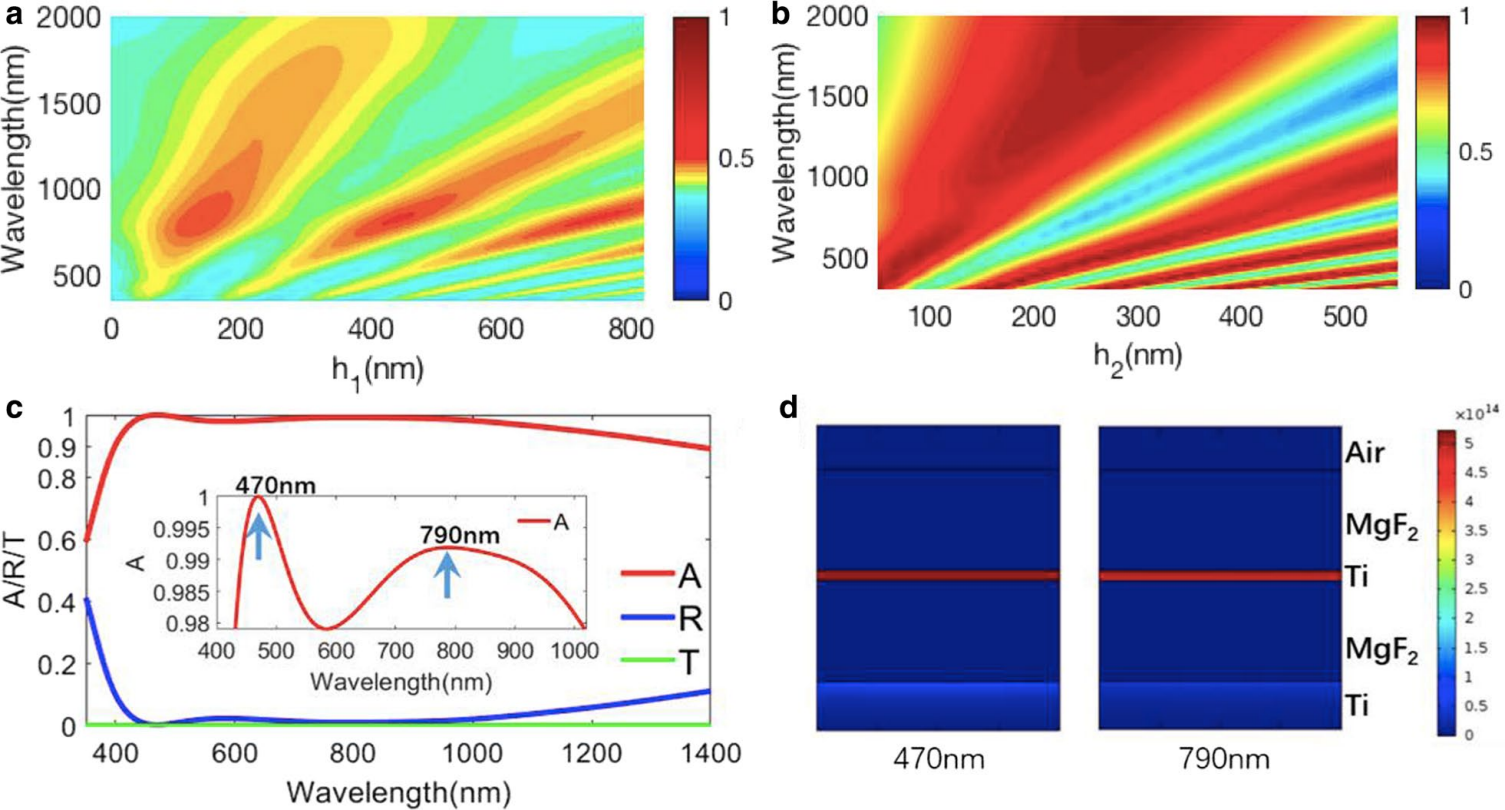
**a** Simulated absorption spectra of the structure of MgF_2_/Ti/MgF_2_ layers with different *h*_1_. **b** Simulated absorption spectra of the the structure of Ti/MgF_2_/Ti layers with different *h*_2_. **c** Simulated absorption/transmission/reflection spectra of the metasurface structure consisting of MgF_2_/Ti/MgF_2_/Ti layers on a substrate. **d** Power dissipation density calculations for the structure at the wavelengths of two absorption peaks

For the IM structure in Fig. 1b, the MgF_2_/Ti planar structure is placed on the MgF_2_ substrate, and the thickness (*h*_*m*_) of the Ti layer is 10 nm. As shown in Fig. 2a, with the increase of the thickness of the dielectric layer, it can be observed the number of resonance modes in the MgF_2_/Ti layers structure gradually increases, agreeing well with Eq. (1). This indicates that the MgF_2_/Ti layers structure in Fig. 1b **is** a resonator [48]. Meanwhile, we also can find that the lower resonance mode (corresponding to the smaller thickness of the dielectric layer) has larger bandwidth (∆*λ*_BW_). For the MIM structure in Fig. 1c, the thickness (*h*_2_) of the top Ti layer is designed to be 10 nm, whereas the bottom Ag is infinite to block the transmitted light. Similarly, we can see the obvious resonance behavior, and the lower-order resonance mode has larger bandwidth (∆*λ*_BW_), as shown in Fig. 2b.

In order to obtain a broadband absorption spectrum, both of the Resonator 1 and the Resonator 2 operate in the lowest-order resonant mode by reasonable selecting the thickness (*h*_1_ = 105 nm, *h*_2_ = 95 nm) of the two dielectric (phase matching) layers. Since the reflectivity of the dielectric-air interface and dielectric-metal interface is relatively low, the fundamental resonant mode has a high optical loss. Figure 2c plots the simulation results of the absorption (red solid line) of the metasurface over the visible and near-infrared wavelengths ranging from 350 to 1500 nm. Due to the existence of two resonators, there are two absorption peaks at a shorter wavelength (around 470 nm) and a longer wavelength (around 790 nm), as shown in Fig. 2c. These two resonant peaks deviate slightly from the resonant peaks of the isolated resonators, because of the interaction of the two resonators. Due to the superimposition of the resonators, the 1D few-layer metasurface has an averaged absorption efficiency higher than 97% at wavelengths of 350–1200 nm. The operating bandwidth (*A* > 90%) of ∆*λ*_BW_ = 1000 nm is greater than those (∆*λ*_BW_ ≤ 750 nm)of the previous solar absorbers based on IM and MIM structures [1–8].

To further verify the physical mechanism of the 1D metasurface absorbers, the maps of power dissipation density distributions at the two absorption peaks are calculated, and the results are depicted in Fig. 2d. As expected, the incident light is mainly absorbed in the thin absorbing (high-loss metal) layer. Moreover, to prove the effectiveness and universality of the proposed structural design, we also simulate the performance of the metasurfaces by other high-loss metals. For example, the simulation results of the absorption, transmission, and reflection of the non-noble metasurfaces by using other metals (such as W, Ni and Cr) are depicted in Additional file 1: Fig. S1. In the simulation, the materials of the first and third layers are MgF_2_. The metasurface by using W also has an average absorption above 97% at wavelengths ranging from 350 to 1000 nm.

**Fig. 3:**
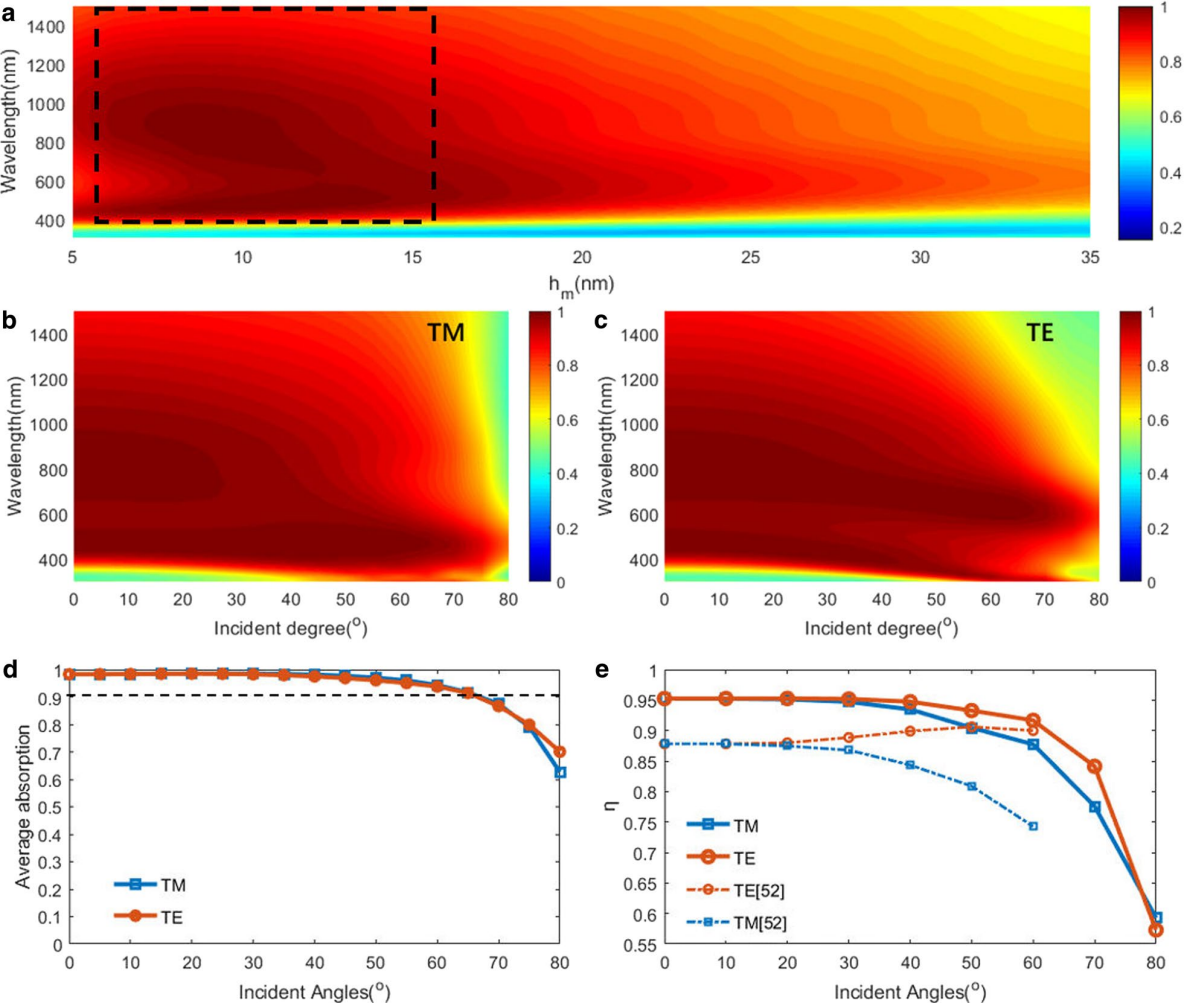
**a** Simulated absorption spectra of the metasurface structure with different *h*_*m*_. **b**–**c** Angle-dependent absorption spectra of the metasurface absorber under **b** TE-polarized and **c** TM-polarized lights, respectively. **d** Averaged absorbance ranging from 350 to 1000 nm at various incident angles from 0° to 80° of TE-polarized and TM-polarized lights. **e** Calculated solar-to-thermal efficiencies (*C* = 1000) at various incident angles from 0° to 80°of TE-polarized and TM-polarized light

The absorption spectra of metasurfaces with different thicknesses of the absorbing layer are calculated and discussed in Fig. 3a. The metasurface absorber maintains its average absorption above 90% at wavelengths of 400-1200 nm within a wide range of the thickness of the thin absorbing layer (6 nm < *d*_m_ < 16 nm). The result indicates a high absorption performance can be achieved in a wide range of the thickness of a thin absorbing layer, which is propitious for convenient fabrication. However, the previous work only using a single resonator requires a high-precision thickness of the thin absorbing layer for a critical coupling condition to achieve an efficient absorption.

The angle and polarization dependences are also an important criterion to evaluate an optical absorber, so we further calculate its absorption spectra under different incident angles for both transverse electric (TE) and transverse magnetic (TM) modes, as shown in Fig. 3b, c. The average absorption at wavelengths ranging from 350 to 1000 nm is also calculated and depicted in Fig. 3d. We can clearly see that the average absorption at wavelengths ranging from 350 to 1000 nm is kept above 90% at an incidence angle as high as 65°. Their average absorption is decreased slightly with the increase of incident angles and is still up to 80% for incident angles up to 75° under TE-polarized and TM-polarized light. For these previous few-layer planar nanostructures based on one resonator, the average absorption efficiency at wavelengths ranging from 400 to 1000 nm would drop below 90% for an incident angle larger than 40° under TE-polarization incidence.[1–8, 48, 50].These results show that this metasurface possessses the best performance of angular independence compared with previous few-layer planar absorbers [1–8]. The reason is that, most previously reported few-layer planar absorbers are based on only one kind of absorption mechanisms. However, the absorption in our absorber is based on the superimposing of two high-loss resonators. Based on the simulated absorption spectra, we calculate the solar-to-thermal conversion efficiency *ƞ*, as follows[52]

2 $$\Delta = {E_{\upalpha }} - {E_R} = \frac{{C \times \smallint {\text{d}}\lambda {\upalpha }\left( \lambda \right){E_{{\text{solar}}}}\left( \lambda \right) - \smallint {\text{d}}\lambda \alpha \left( \lambda \right){E_{\text{B}}}\left( \lambda \right)}}{{C \times \smallint {\text{d}}\lambda {E_{{\text{solar}}}}\left( \lambda \right)}}$$

where *E*_α_ is the total solar absorbance; *E*_R_ is the thermal radiation loss; *E*_solar_ is the spectral solar irradiation; E_B_ (*λ*,*T*_A_) is the blackbody radiation at temperature *T*_A_; and C is the concentration factor that is usually on the order of 1 to 1000[52]. The calculated results are displayed by the solid lines in Fig. 3e. The absorber performs high *ƞ*_solar thermal_ of > 0.9 under a TE-polarized light with an incident angle of *θ* <  = 60°, as shown in Fig. 3e. Meanwhile, the absorber remains *ƞ* >  = 0.9 under a TM-polarized light with an incident angle of *θ* <  = 55°, as shown in Fig. 3e. This performance is better than that of previous solar absorbers [52]. *ƞ* with various incident angles in Ref. [52] is depicted by the dotted line in Fig. 3e. For TM polarization, the *ƞ* of our absorber is about 20% higher than that of the absorber in [52]. These results reveal that the optical absorption of our metasurface is not only broadband but also wide-angle.

## Experimental Results and Discussion

**Fig. 4:**
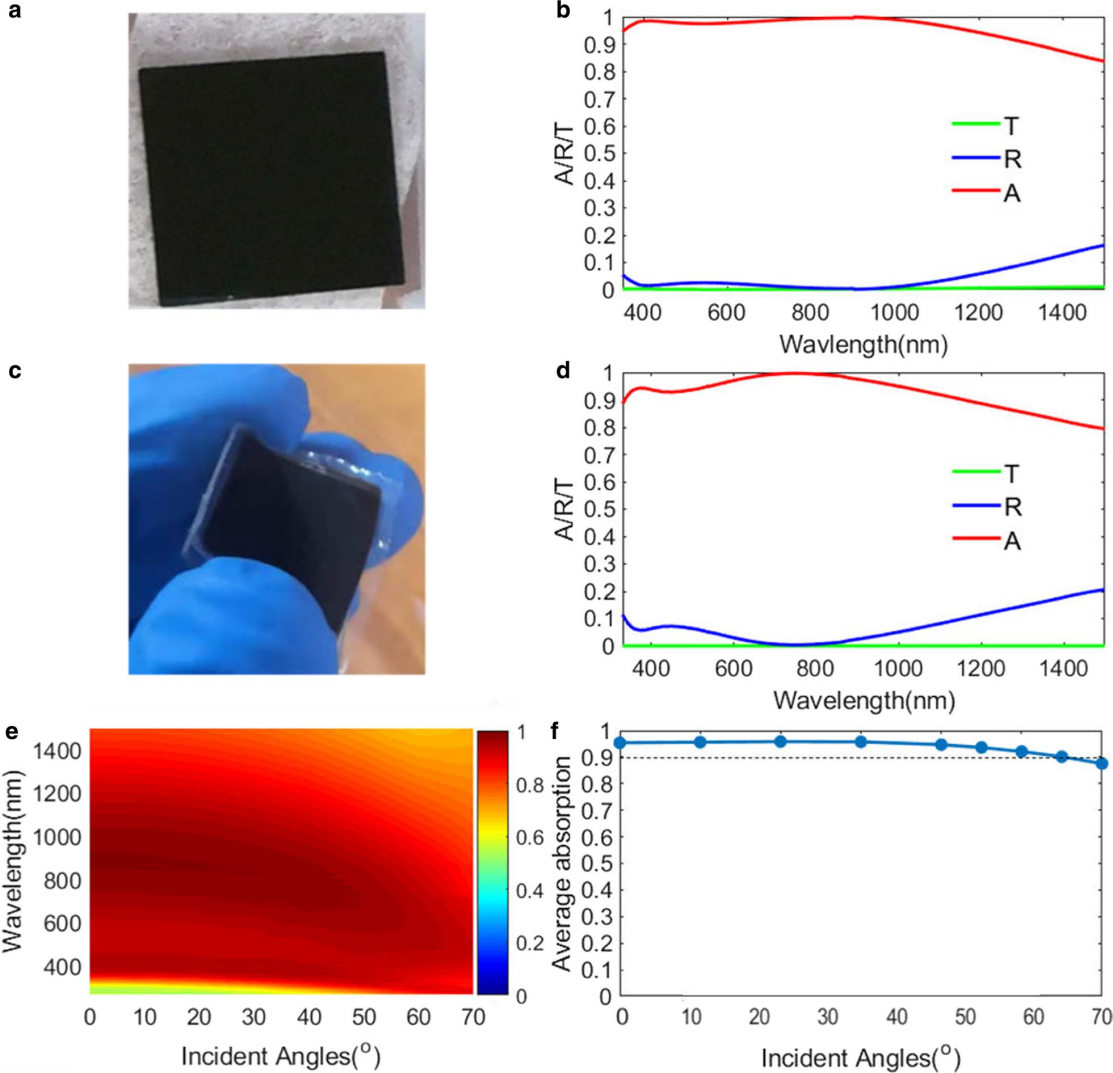
**a** Photograph of the metasurface on a glass substrate. **b** Experimental absorption/transmission/reflection spectra of a metasurface structure. **c** Photograph of a flexible metasurface on a PE substrate. **d** Experimental absorption/transmission/reflection spectra of a flexible metasurface. **e** Experimental angle-dependent absorption spectra of a metasurface absorber under an unpolarized light. **f** Experimental averaged absorption at wavelengths ranging from 350 to 1000 nm at various incident angles from 0° to 70°of a unpolarized light

To validate the proposed 1D metasurface absorber, we fabricate the designed metasurface by only using an E-beam evaporator. The bottom layer of Ti (150 nm), a spacer of MgF_2_ (95 nm), a thin absorption Ti layer (10 nm), and a MgF_2_ layer (105 nm) are deposited on a glass substrate. The image of the fabricated absorber is depicted in Fig. 4a, and we can observe that the sample is all black. Next, the optical transmission (*T*) and reflection (R) spectra of the metasurface are measured at wavelengths of 350–1500 nm with a Shimadzu UV3600 spectrophotometer attached to the integrating sphere (ISR-3100). The absorption (*A*) is then calculated by *A* = 1–*R* –*T*. Clearly, we see a broadband absorption spectrum with two absorption peaks, exhibiting a good agreement between simulation results in Fig. 2c and experiment results in Fig. 4b. The average absorption of the experiment results is above 97% at wavelengths from 350 to 1200 nm. The BW (∆*λ*_BW_) of the absorption larger than 90% is up to 1030 nm (350 nm-1380 nm), which is greater than that (∆*λ*_BW_ = 750 nm [51]) of previously reported IM and MIM planar absorber [2–17].

Moreover, we also deposit the metasurface structure on a flexible (PE, polyethylene) substrate, and Fig. 4c represents the image of the fabricated flexible sample, which is also black. The optical properties of the flexible sample are also measured and depicted in Fig. 4d, and an average absorption above 95% at wavelengths of 350–1100 nm is obtained. The reason of the small absorption difference at shorter wavelengths between Fig. 4b, d is that it is a little difficult to ensure its high-precision thickness of metal/dielectric in deposition processes. As shown in Fig. 4e, we also measure the absorption spectra under different incident angles with unpolarized light. The experiment results show that our absorber is insensitive to the incident angle, which is consistent with the simulation results. The measured average absorption ranging from 350 to 1000 nm at various incident angles from 0° to 70° is also depicted in Fig. 4f.The measured average absorption at wavelengths ranging from 350 nm-1000 nm is kept above 90% at incidence angle as high as 65°, which is in good agreement with the simulation result in Fig. 3d. Note that, for these reported few-layer planar nanostructures based on one resonator, the average absorption efficiency at wavelengths ranging from 400 to 1000 nm would drop below 90% for incident angles larger than 40° under TE-polarization incidence.[1–8, 48, 50]

**Fig. 5:**
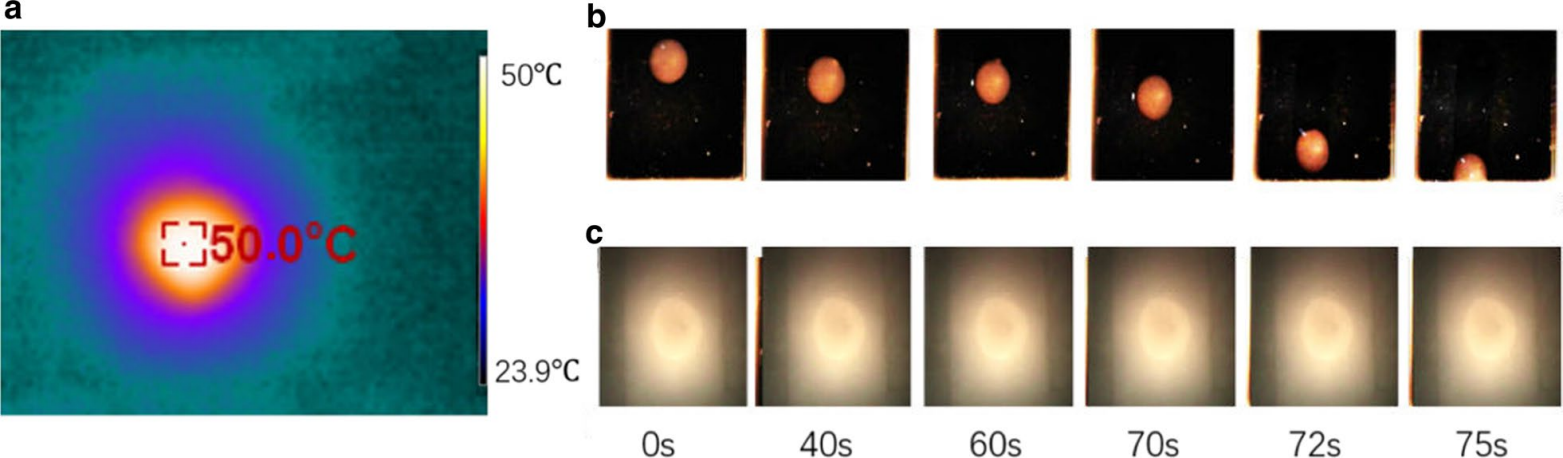
**a** Thermal image of a metasurface absorber. **b** Representative snapshots of a frozen water droplet on an illuminated metasurface and glass

For further evaluating the potential of our metasurface in photo-thermal applications, we also characterize its light-heating property. We use a broadband halogen light source, and then record the increased temperature of a metasurface sample by a XINTEST-HT18 infrared thermometer. The power of the halogen light source is measured by a XINBAO-SM206 photometer in the following experiment. From Fig. 5a, it can be clearly seen that the generated heats are highly confined around the metasurface sample. The flexible metasurface increases its surface temperature by 25.1 K from room temperature under the halogen light of *P* = 1.2 kW/m^2^. The increase of surface temperature is higher than that of the recently reported solar absorbers based on a gold-particle metasurface (*A* = 83%, ∆*T*_e_ = 12 °C, *P* = 2.4 kW/m^2^)[54] and gold/nickel plasmonic metasurface (∆*T*_e_ = 8 °C, *P* = 1kw/m^2^) [55] Furthermore, Fig. 5b, c shows representative image sequences of a frozen water droplet on a metasurface and glass samples. Firstly, a single water droplet is deposited and frozen on the surface of a metasurface and glass. Then, a halogen lamp light (*P* ≈ 1.2 kW/m^2^) illuminates the surface with the frozen droplet adhered to the metasurface or glass. For the metasurface sample, the droplet starts sliding after 40 s, and it is fully removed within approximately 75 s. In contrast, no changes of the frozen droplet are seen for the glass under the same illumination. Note that, the illumination intensity (*P* = 1.2 kW/m^2^) of the incident light in our work is only half of that (*P* = 2.4 kW/m^2^) in previous solar anti-ice studies based on gold/TiO_2_ particle metasurfaces[54], indicating that our metasurface is more advantageous to practical applications.

## Conclusions

In summary, an efficient design strategy was proposed to achieve broadband absorbers based on 1D non-noble metasurface, consisting of dielectric/metal/dielectric/ metal layers. Owing to the superimposing of two high-loss resonators, an average absorption above 97% at wavelengths of 350–1200 nm was achieved. The bandwidth of the absorption larger than 90% was up to 1000 nm (410–1410 nm), which was greater than that (≤ 750 nm) of previous MIM planar absorbers [1, 5, 8, 25–27]. The metasurface was fabricated by a simple E-beam deposition method, providing the possibility of large-area applications. The simulation and experiment results showed that the broadband absorption of our absorbers was kept above 90% at an incidence angle as high as 65° ranging from 350 to 1000 nm. For previous few-layer planar absorbers, the average absorption efficiency at wavelengths ranging from 400 to 1000 nm would drop below 90% for an incident angle larger than 40° under a TE-polarization incidence.[1–8, 48, 50].Additionally, the flexibility was also demonstrated by deposit the metasurface on a flexible substrate. The flexible metasurface increased its surface temperature by 25.1 K from room temperature under a halogen lamp of *P* = 1.2 kW/m^2^. For practical applications, we investigated the capability of the flexible metasurface for removing ice under a halogen lamp of *P* = 1.2 kW/m^2^. This 1D metasurface with broadband and efficient absorption might have potential applications in solar-energy-driven icephobicity.

## Supplementary information

**Additional file 1: Fig. S1**. Simulated absorption for the metasurface absorbers consisting of MgF2/W/MgF2/W, MgF2/Ni/MgF2/Ni, and MgF2/Cr/MgF2/Cr layers, respectively.

## Data Availability

The datasets generated during and/or analyzed during the current study are available from the corresponding authors on reasonable request.

## Abbreviations

BW: bandwidths
FDTD: finite-difference time domain
IM: insulator–metal
MIM: metal–insulator–metal
