# Supplementary material for: Broadening Bandwidths of Few-Layer Absorbers by Superimposing Two High-Loss Resonators
Source: Nanoscale Res Lett. 2021 Feb 10;16:26. doi: 10.1186/s11671-020-03471-1 (PMC7876209; doi:10.1186/s11671-020-03471-1)
Supplement: Supplementary file 1 — Additional file 1: Fig. S1. Simulated absorption for the metasurface absorbers consisting of MgF2/W/MgF2/W, MgF2/Ni/MgF2/Ni, and MgF2/Cr/MgF2/Cr layers, respectively. [file 11671_2020_3471_MOESM1_ESM.docx]

Supporting Information

**Broadening bandwidths of few-layer absorbers by superimposing two high-loss resonators**

Dong Wu ^a^, Jianjun Chen *^a,b,c,d,e^

a. State Key Laboratory for Mesoscopic Physics, School of Physics, Peking University, Beijing, 100871, China.

b. Department of Physics and Applied Optics Beijing Area Major Laboratory, Beijing Normal University, Beijing 100875, China

c. Peking University Yangtze Delta Institute of Optoelectronics, Nantong 226010, Jiangsu, China.

d. Frontiers Science Center for Nano-optoelectronics & Collaborative Innovation Center of Quantum Matter, Peking University, Beijing, 100871, China.

e. Collaborative Innovation Center of Extreme Optics, Shanxi University, Taiyuan, Shanxi, 030006, China.

Email: jjchern@pku.edu.cn


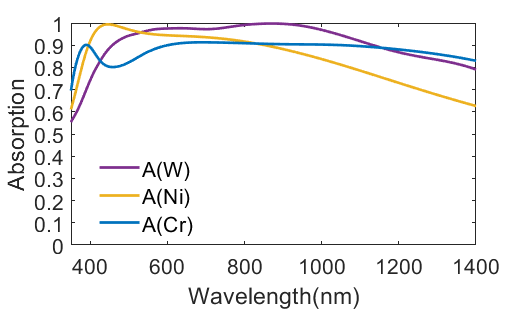


Fig.S1 Simulated absorption for the metasurface absorbers consisting of MgF_2_/W/MgF_2_/W, MgF_2_/Ni/MgF_2_/Ni, and MgF_2_/Cr/MgF_2_/Cr layers, respectively.
